# Hybrid Technique on the Total Arch Replacement for Type A Aortic Dissection: 12-year Clinical and Radiographical Outcomes From a Single Center

**DOI:** 10.3389/fcvm.2022.820653

**Published:** 2022-02-28

**Authors:** Bowen Zhang, Xiaogang Sun, Yanxiang Liu, Yaojun Dun, Shenghua Liang, Cuntao Yu, Xiangyang Qian, Haoyu Gao, Jie Ren, Luchen Wang, Sangyu Zhou

**Affiliations:** Department of Cardiovascular Surgery, National Center for Cardiovascular Diseases, Fuwai Hospital, Chinese Academy of Medical Sciences and Peking Union Medical College, Beijing, China

**Keywords:** type A aortic dissection, total arch replacement, hybrid technique, debranching, endovascular, hypothermia, aortic remodeling

## Abstract

**Objective:**

Hybrid total arch replacement (HTAR) was an alternative for type A aortic dissection (TAAD). This study aimed to evaluate the clinical and radiographical outcomes of HTAR for TAAD and to evaluate the clinical outcomes of performing this procedure under mild hypothermia.

**Methods:**

A total of 209 patients who underwent HTAR for TAAD were retrospectively analyzed and stratified into mild (*n* = 48) and moderate (*n* = 161) hypothermia groups to evaluate the effects of mild hypothermia on the clinical outcomes. Long-term clinical outcomes were evaluated by the overall survival and adverse aortic events (AAEs). A total of 176 patients with preoperative and at least one-time postoperative aortic computed tomography angiography in our institute were included for evaluating the late aortic remodeling (aortic diameter and false lumen thrombosis).

**Results:**

The median follow-up period was 48.3 (interquartile range [IQR] = 28.4–73.7) months. The overall survival rate was 88.0, 83.2, and 77.1% at the 1, 5, and 10 years, respectively, and in the presence of death as a competing risk, the cumulative incidence of AAEs was 4.8, 9.9, and 12.1% at the 1, 5, and 10 years. The aortic diameters were stable in the descending thoracic and abdominal aorta (*P* > 0.05 in all the measured aortic segments). A total of 100% complete false lumen thrombosis rate in the stent covered and distal thoracic aorta were achieved at 1 year (64/64) and 4 years (18/18), respectively after HTAR. The overall composite adverse events morbidity and mortality were 18.7 and 10.0%. Mild hypothermia (31.2, IQR = 30.2–32.0) achieved similar composite adverse events morbidity (mild: 14.6 vs. moderate: 19.9%, *P* = 0.41) and early mortality (mild: 10.4 vs. moderate: 9.9%, *P* = 1.00) compared with moderate hypothermia (median 27.7, IQR = 27–28.1) group, but mild hypothermia group needed shorter cardiopulmonary bypass (mild: 111, IQR = 93–145 min vs. moderate: 136, IQR = 114–173 min, *P* < 0.001) and aortic cross-clamping (mild: 45, IQR = 37–56 min vs. moderate: 78, IQR = 54–107 min, *P* < 0.001) time.

**Conclusion:**

Hybrid total arch replacement achieved desirable early and long-term clinical outcomes for TAAD. Performing HTAR under mild hypothermia was as safe as under moderate hypothermia. After HTAR for TAAD, dissected aorta achieved desirable aortic remodeling, presenting as stable aortic diameters and false lumen complete thrombosis. In all, HTAR is a practical treatment for TAAD.

## Introduction

Type A aortic dissection (TAAD) remains a catastrophic event with substantial morbidity and mortality, despite advances in surgical technique and perioperative care (1). Extensive aortic arch repair for TAAD has been gradually accepted for TAAD (2, 3). For extensive aortic arch repair, conventional total arch replacement (TAR) has a long history and has achieved desirable long-term outcomes (4), but its huge surgical invasion remained a non-negligible problem. Endovascular total arch repair is a new technique. Although it reduced operative mortality, its long-term outcomes were unclear (5). Besides, it also requires a solid foundation of interventional surgery. Hybrid total arch replacement (HTAR) that combined ascending aorta replacement, supra-arch debranching, and stent-graft deployment to exclude the entire lesioned aortic arch seemed to be a practical extensive arch repair strategy for TAAD (6). Previous studies about the early and long-term outcome of HTAR for extensive arch disease distinguished from centers (7–11). And these reports rarely had a large sample size and rarely focused on TAAD only. Besides, the late aortic remodeling was also rarely reported by previous studies. HTAR has a 12-year history and is a practical treatment for TAAD in our institute, so the first objective was to summarize the 12-year experience of HTAR for TAAD and evaluate its clinical and radiographic outcomes.

In the aortic arch surgery, moderate-to-deep hypothermia was widely accepted as an organ-protective factor when circulation was arrested (12). Distal circulatory arrest and prolonged unilateral cerebral perfusion are theoretically avoided in the HTAR, it deserves to reconsider whether mild hypothermia is enough for organ protection. Previous studies reported that mild hypothermia achieved not inferior outcomes compared with moderate hypothermia in nonextensive arch repair (13). But there were few reports about mild hypothermia in extensive arch repair. In our institute, mild hypothermia was attempted when performing HTAR for TAAD, therefore, the second objective of this study was to evaluate the effects of mild hypothermia on the clinical outcomes of HTAR for TAAD.

## Patients and Methods

### Study Cohort

**Figure 1:**
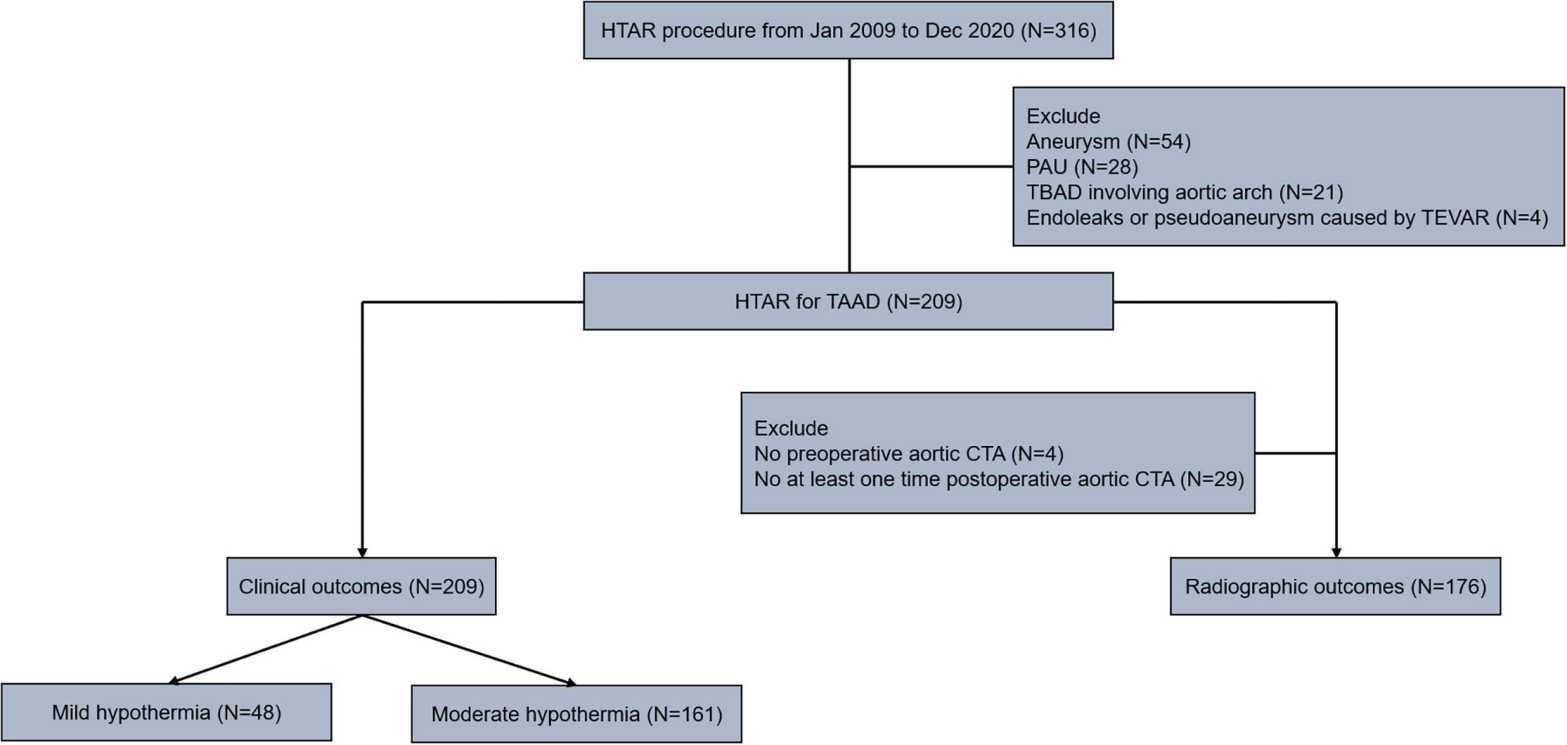
Flow diagram of the study cohort. Two hundred and nine patients who underwent HTAR for TAAD from Jan 2009 to December 2020 were retrospectively analyzed. For analyzing the clinical outcomes, all patients were stratified into mild (*N* = 48) and moderate (*N* = 161) hypothermia group depending on the lowest nasopharyngeal temperature (30°C). One hundred and seventy six patients who underwent preoperative and at least one-time postoperative aortic CTA examination in our institute simultaneously were included for analyzing the radiographic outcomes. CTA, computed tomography angiography; HTAR, hybrid total arch replacement; TAAD, type A aortic dissection.

This study was approved by the ethics committees of Fuwai hospital (No. 2021-1557) and written informed consent was waived. Fuwai hospital medical system identified 316 patients who underwent HTAR from January 2009 to December 2020. Excluding 107 patients who were not diagnosed with TAAD (54 aneurysm, 28 penetrating aortic ulcers, 21 type B aortic dissection involving aortic arch and 4 endoleaks or pseudoaneurysm caused by thoracic endovascular aortic repair), a total of 209 patients diagnosed with TAAD and underwent HTAR were analyzed retrospectively for clinical outcomes evaluation. When analyzed the effect of mild hypothermia on the clinical outcomes, all patients were stratified into two groups depending on the lowest nasopharyngeal temperature (30°C), 48 patients in the mild hypothermia group and 161 patients in the moderate hypothermia group. For the radiographic outcome evaluation, excluding four patients without preoperative aortic computed tomography angiography (CTA) examination in our institute and 29 patients without at least one-time postoperative aortic CTA examination in our institute, 176 patients who had preoperative aortic CTA images and at least one-time postoperative aortic CTA images in our institute simultaneously were included for radiographic analysis. The patient flow diagram is shown in Figure 1.

### Surgical Indication

Generally, conventional total arch replacement with frozen elephant trunk (TAR with FET) procedure was still an optimal option for TAAD in our institute, HTAR was considered as an alternative when the patients were unsuitable for TAR with FET, and mainly used in the following conditions for TAAD:

1. Old patients (age > 65 years) or with multiple comorbidities: because of high-risk of circulatory arrest and prolonged unilateral cerebral perfusion in these patients.
2. Huge intimal tears or huge false lumen located at the distal arch: because if performing FET procedure, distal aortic arch anastomosis was difficult in this condition.
3. Middle-to-distal descending aorta existed huge intimal tears: the FET procedure was too short to cover them at this region, which impacted the false lumen thrombosis process.

For the choice of hypothermia, in our institute, the lowest nasopharyngeal temperature when performing extensive aortic arch surgery was mainly set at 24–28°C depending upon different operative techniques. A senior surgeon attempted to use mild hypothermia in HTAR for TAAD. However, there was no strict indication, which was mainly dependent on choices of the surgeons. The temperature setting was also in the exploratory stage, and the temperature was continually adjusted with cases accumulation.

### Surgical Technique and Postoperative Treatment

**Figure 2:**
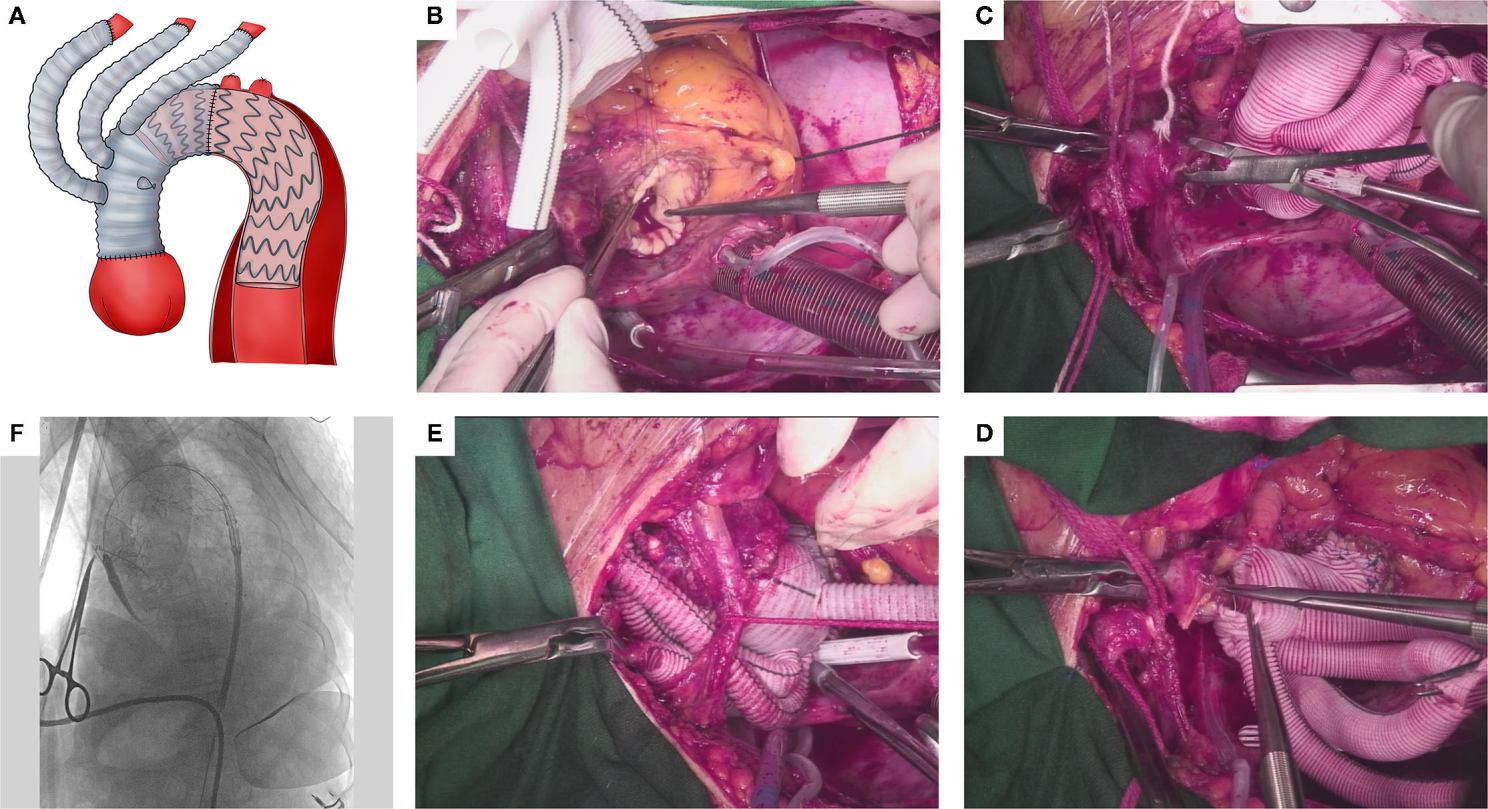
Operative technique of HTAR. **(A)** Overall schematic diagram of HTAR: ascending aorta replacement, total supra-arch debranching and stent graft deployment to exclude the entire lesioned aortic arch. **(B)** Proximal anastomosis: the proximity of tetrafurcate vascular prosthesis graft was anastomosed to the sinotubular junction (aortic root has been repaired) by running suture with prolene. **(C)** Clamp site change: one aortic clamp was set on the aorta between the innominate artery and LCCA and another clamp was set on the innominate artery. The former aortic clamp on the ascending aorta could be removed. **(D)** Distal anastomosis: the distal anastomosis was made before the level of LCCA to ensure a sufficient proximal landing zone. **(E)** Supra-arch debranching: LCCA, LSCA and innominate artery were reconstructed by end-to-end anastomosis sequentially. **(F)** Endovascular portion: deploying a stent graft to exclude the entire lesioned aortic arch. HTAR, hybrid total arch replacement; LCCA, left common carotid artery; LSCA, left subclavian artery.

The operative technique was reported in the previous studies (14) and the schematic diagram is also shown in Figure 2A. All the procedures were performed in the hybrid operating room and under general anesthesia. HTAR in our institute was one-stage and divided into two portions, open repair and endovascular repair portion.

#### Open Repair Portion

After median sternotomy, visualization of supra-arch branches, and systemic heparinization, patients were placed on cardiopulmonary bypass (CPB) by cannulation of the right axillary artery, right femoral artery, and the right atrium. Then, cross-clamped the ascending aorta made a longitude incision in the ascending aorta and perfused cardioplegia solutions antegrade. If the aortic root was involved by the dissection, it should be repaired or replaced primarily. The tetrafurcate vascular prosthesis graft (Hemashield Platinum, Maquet Cardiovascular LLC, New Jersey, USA) was used for supracommissural ascending aortic replacement, and it was first anastomosed to the sinotubular junction (Figure 2B). There was a change in the clamp site before we could manage the distal graft anastomosis (Figure 2C). One aortic clamp was set on the aorta between the innominate artery and left common carotid artery (LCCA), another clamp was set on the innominate artery. The former clamp on the ascending aorta could be removed and the dissected aortic tissue proximal to the aortic clamp was resected. The distal anastomosis was made just proximal to the aortic clamp (Figure 2D). Then, removed the aortic clamp (the innominate artery was still clamped) and reconstructed the LCCA. After LCCA reconstruction, rewarming started. During the rewarming process, the left subclavian artery and the innominate artery were reconstructed sequentially (Figure 2E).

#### Endovascular Repair Portion

Angiography was performed to measure the length and diameter of the proximal landing zone (Figure 2F). In the early stages of performing HTAR, antegrade access through 10 mm branches of the tetrafurcate vascular prosthesis was more common. With the familiarity of interventional techniques, retrograde access through the femoral artery was a routine access. The size and length of stent grafts were chosen depending on the image analysis. The diameter of the stent was usually oversized 10–20% by the proximal landing zone and the distal end of the stent was at least located at the descending aorta of the 6th thoracic vertebra or more distal. Brands of commercially available stents included Zenith (Cook Medical Inc., Bloomington, IN, USA), Relay (Bolton Medical, Sunrise, Florida, USA), Talent and Valiant (Medtronic Inc., Santa Rosa, California, USA), Gore stent graft (WL Gore & Association, Flagstaff, Arizona, USA) and Hercules (MicroPort Endovascular MedTech Co., Ltd., Shanghai, China).

Hemostasis, indwelling drainage, and closure completed the surgery and the patient was transferred to the intensive care unit (ICU). After several days of intensive care and recovery, the patients were discharged and were recommended to undergo aortic CTA examination at 3 months after the operation and annually thereafter.

### Outcome Criteria

The primary endpoints were defined as long-term clinical and radiographical outcomes. The long-term clinical outcomes were evaluated by overall survival, adverse aortic events (AAEs). Of those, AAEs were defined as sudden aortic rupture, aortic-related reintervention, pseudoaneurysm, endoleaks, distal stent-induced new entry (SINE), graft infection, debranching graft occlusion, etc. The long-term radiographic outcomes were evaluated by the aortic remodeling, including the aortic diameter change and false lumen thrombosis measurement. Aortic diameter measurements were made at the following four segments: pulmonary artery bifurcation level (represented the proximal descending aorta and was typically covered by the stent), Th10 (10th thoracic vertebrae) level (represented the middle to the distal descending aorta and was usually not covered by the stent), the celiac artery level (measured at the region above the celiac artery and represented the proximal abdominal aorta), and the renal artery level (measured at the region below the renal artery and represented the distal abdominal aorta). False lumen thrombosis measurements were made in three segments of the aorta: stent covered thoracic aorta, stent distal thoracic aorta, and abdominal aorta. Complete thrombosis was defined as the absence of contrast in the false lumen.

The secondary endpoints were defined as early clinical outcomes, which were evaluated by the early composite adverse events (including early mortality, low cardiac output syndrome (LCOS), malignant ventricular arrhythmia, stroke, paraplegia, hemodialysis, respiratory failure, and unplanned reoperation), and other early complications (including acute kidney injury, hepatic dysfunction, temporary neurologic deficit, mechanical ventilation time, ICU stays, and postoperative hospital stays). The detailed definition of the early clinical outcomes is shown in Supplemental Table 1.

### Statistical Analysis

Descriptive statistics for categorical variables were reported as the frequency and percentage, whereas continuous variables were reported as the mean ± SD or median with interquartile range (IQR) depending upon a normal distribution. Categorical variables were compared with Pearson chi-squared test or Fisher's exact test, and continuous variables were compared with Student's *t*-test or Mann–Whitney *U* test as appropriate.

Multivariable logistic regression models were used to find the independent risk factors for the early major complications. All the potential covariates of interest were included in a univariable logistic regression model. The multivariable logistic regression model included significant variables (*P* < 0.1) in the univariate logistic regression.

Overall long-term survival was estimated by the Kaplan–Meier method combined with the log-rank test. AAEs were evaluated by using competing risk regression analysis (Fine & Gray model) and death served as the competing risk.

The comparison of aortic diameter changes and complete false lumen thrombosis during follow-up was performed using the repeated measures ANOVA.

For all analyses, a *P* < 0.05 was considered as statistically significant, and all the statistical tests were two-sided. Statistical analyses were performed using SPSS, version 21.0 (SPSS Incorporation, Chicago, Illinois, USA) and R version 4.0.4 (The R Foundation for Statistical Computing, Vienna, Austria).

## Results

### Baseline Characteristics

The median age of the entire cohort was 62 (IQR: 57–67) years and 129 (61.7%) were male. A total of 196 (93.8%) patients were diagnosed with acute TAAD and 139 (66.5%) patients underwent emergency operation. Malperfusion syndrome occurred in 43 (20.6%) patients, of those, cerebral, myocardial, mesenteric, renal, and lower extremity malperfusion occurred at 17 (8.1%), 7 (3.3%), 10 (4.8%), 6 (2.9%), and 9 (4.3%) patients, respectively. When divided all the patients into the mild and moderate hypothermia groups, the baseline characteristic between the two groups was equal, which is shown in Table 1.

**Table 1:** Baseline characteristic.

**Variables**	**All patients** **(*n* = 209)**	**Mild hypothermia** **(*n* = 48)**	**Moderate hypothermia** **(*n* = 161)**	***P* Value**
Age, y, mean (SD)	62 (57–67)	62 (56– 68)	63 (58– 67)	0.48
Male sex *n*, (%)	129 (61.7)	30 (62.5)	99 (61.5)	0.90
BMI, kg/m^2^, median (IQR)	25.3 (23.4– 27.8)	25.9 (24.0– 27.8)	25.1 (23.1– 27.7)	0.47
Acute TAAD *n*, (%)	196 (93.8)	45 (93.8)	151 (93.8)	0.99
Emergency operation *n*, (%)	139 (66.5)	30 (62.5)	109 (67.7)	0.50
Medical history				
Hypertension *n*, (%)	192 (91.9)	46 (95.8)	146 (90.7)	0.40
Coronary artery disease *n*, (%)	36 (17.2)	6 (12.5)	30 (18.6)	0.32
Diabetes mellitus *n*, (%)	12 (5.7)	4 (8.3)	8 (5.0)	0.60
Peripheral arterial disease *n*, (%)	13 (6.2)	2 (4.2)	11 (6.8)	0.74
Old cerebral vessel accidents *n*, (%)	20 (9.6)	5 (10.4)	15 (9.3)	1.00
Chronic kidney disease *n*, (%)	7 (3.3)	3 (6.3)	4 (2.5)	0.42
Chronic obstructive pulmonary disease *n*, (%)	9 (4.3)	1 (2.1)	8 (5.0)	0.65
Smoking *n*, (%)	78 (37.3)	22 (45.8)	56 (34.8)	0.17
Malperfusion syndrome *n*, (%)	43 (20.6)	10 (20.8)	33 (20.5)	0.96
Cerebral *n*, (%)	17 (8.1)	4 (8.3)	13 (8.1)	1.00
Myocardial *n*, (%)	7 (3.3)	2 (4.2)	5 (3.1)	1.00
Mesenteric *n*, (%)	10 (4.8)	3 (6.3)	7 (4.3)	0.88
Renal *n*, (%)	6 (2.9)	0 (0)	6 (3.7)	0.39
Lower extremity *n*, (%)	9 (4.3)	2 (4.2)	7 (4.3)	1.00
NYHA ≥ Grade III *n*, (%)	7 (3.3)	2 (4.2)	5 (3.1)	1.00
LVEF %, median (IQR)	60 (59– 63)	60 (60– 63)	60 (59– 63)	0.84
Previous sternotomy *n*, (%)	4 (1.9)	0 (0)	4 (2.5)	0.58
Preoperative increasing Scr *n*, (%)	57 (27.3)	15 (31.3)	46 (26.1)	0.48
Preoperative increasing hepatic enzyme *n*, (%)	41 (19.6)	35 (21.7)	6 (12.5)	0.16

*BMI, body mass index; IQR, interquartile range; LVEF, left ventricular ejection fraction; NYHA, New York Heart Association; Scr: serum creatinine; TAAD, type A aortic dissection*.

### Intraoperative Data

The intraoperative data were shown in Table 2 in detail. A total of 48 (23.0) patients underwent an intraoperative concomitant operation for primary heart disease or the aortic root dissection involvement. The median lowest nasopharyngeal and bladder temperatures were 27.7 (IQR: 27–28.1) and 28.9 (IQR: 28.0–29.4) in the moderate hypothermia group and were 31.2 (IQR: 30.2–32.0) and 32.3 (IQR: 31.9–33.3) in the mild hypothermia group. The median CPB and aortic cross-clamping time was 133 (IQR: 105–167) and 66 (IQR: 48–100) min for all the patients and the mild hypothermia group had significant short CPB (mild: 111, IQR = 93~145 min vs. moderate: 136, IQR = 114–173 min, *P* < 0.001) and aortic cross-clamping (mild: 45, IQR = 37–56 min vs. moderate: 78, IQR = 54–107 min, *P* < 0.001) time. A total of 305 stents [one stent: 115 (55.0%), one stents: 92 (44.0%), three patients: 2 (1.0%)] used in the entire cohort, and 84.7% patients accepted retrograde stents deployment. The median oversize of proximity was 14.3% (IQR: 13.3–20.0%) and the distal landing zone was mainly located at upper (45.0%) and middle (47.8%) thoracic regions. The median intraoperative erythrocyte, plasma, and platelet transfusion were 4 (IQR = 0–6) IU, 400 (IQR = 0–600) ml, and 1 (IQR = 1–2) IU.

**Table 2:** Intraoperative characteristic.

**Variables**	**All patients** **(*n* = 209)**	**Mild hypothermia** **(*n* = 48)**	**Moderate hypothermia** **(*n* = 161)**	***P* Value**
Concomitant procedures *n*, (%)	48 (23.0)	9 (18.8)	39 (26.0)	0.10
Valsalva sinus repair *n*, (%)	97 (46.4)	28 (58.3)	69 (42.9)	0.06
Root replacement *n*, (%)	23 (11.0)	5 (10.4)	18 (11.2)	0.88
Coronary artery bypass graft *n*, (%)	34 (16.3)	6 (12.5)	28 (17.4)	0.42
Aortic valve replacement *n*, (%)	6 (2.9)	0 (0)	6 (3.7)	0.34
Others *n*, (%)	3 (1.4)	0 (0)	3 (1.9)	1.00
Lowest nasopharyngeal temperature, °C median (IQR)	28.0 (27.1–28.4)	31.2 (30.2–32.0)	27.7 (27–28.1)	<0.001
Lowest bladder temperature, °C, median (IQR)	29.0 (28.2–30.7)	32.3 (31.9–33.3)	28.9 (28.0–29.4)	<0.001
CPB time, min, median (IQR)	133 (105–167)	111 (93–145)	136 (114–173)	<0.001
Aortic cross-clamping time, min, median (IQR)	66 (48–100)	45 (37–56)	78 (54–107)	<0.001
Operation time, h, median (IQR)	6.5 (5.5–7.5)	6.3 (6.0–7.0)	6.5 (5.5–7.5)	0.31
Delivery approach				0.54
Retrograde *n*, (%)	177 (84.7)	42 (87.5)	135 (83.9)	
Antegrade *n*, (%)	32 (15.3)	6 (12.5)	26 (16.1)	
Number of stents				<0.001
1 *n*, (%)	115 (55.0)	37 (77.1)	78 (48.4)	
2 *n*, (%)	92 (44.0)	11 (22.9)	81 (50.3)	
3 *n*, (%)	2 (1.0)	0 (0)	2 (1.2)	
Oversize of proximity	14.3 (13.3–20.0)	13.3 (13.3–17.6)	14.3 (13.3–21.4)	0.08
<10% *n*, (%)	10 (4.8)	1 (2.1)	9 (5.6)	
10–20% *n*, (%)	149 (71.3)	39 (81.3)	110 (68.3)	
>20% *n*, (%)	50 (23.9)	8 (16.7)	42 (26.1)	
Distal landing zone				0.005
Upper thoracic region^a^ *n*, (%)	94 (45.0)	65 (40.4)	29 (60.4)	
Middle thoracic region^b^ *n*, (%)	100 (47.8)	81 (50.3)	19 (39.6)	
Lower thoracic region^c^ *n*, (%)	15 (7.2)	15 (9.3)	0 (0)	
Intraoperative transfusion *n*, (%)				
Erythrocyte, IU, median (IQR)	4 (0–6)	3 (0–6)	4 (0–6)	0.79
Plasma ml, median (IQR)	400 (0–600)	200 (0–600)	400 (0–600)	0.87
Platelet, IU, median (IQR)	1 (1–2)	2 (1–2)	1 (1–1)	<0.001

*CPB, cardiopulmonary bypass; IQR, interquartile range*.

a *upper thoracic region, the descending aorta from Th6 to Th7*;

b *middle thoracic region, the descending aorta from Th8 to Th10*;

c *lower thoracic region, the descending aorta from Th11 to Th12*.

### Primary Endpoints: Long-Term Clinical and Radiographical Outcomes

#### Long-Term Clinical Outcomes

**Figure 3:**
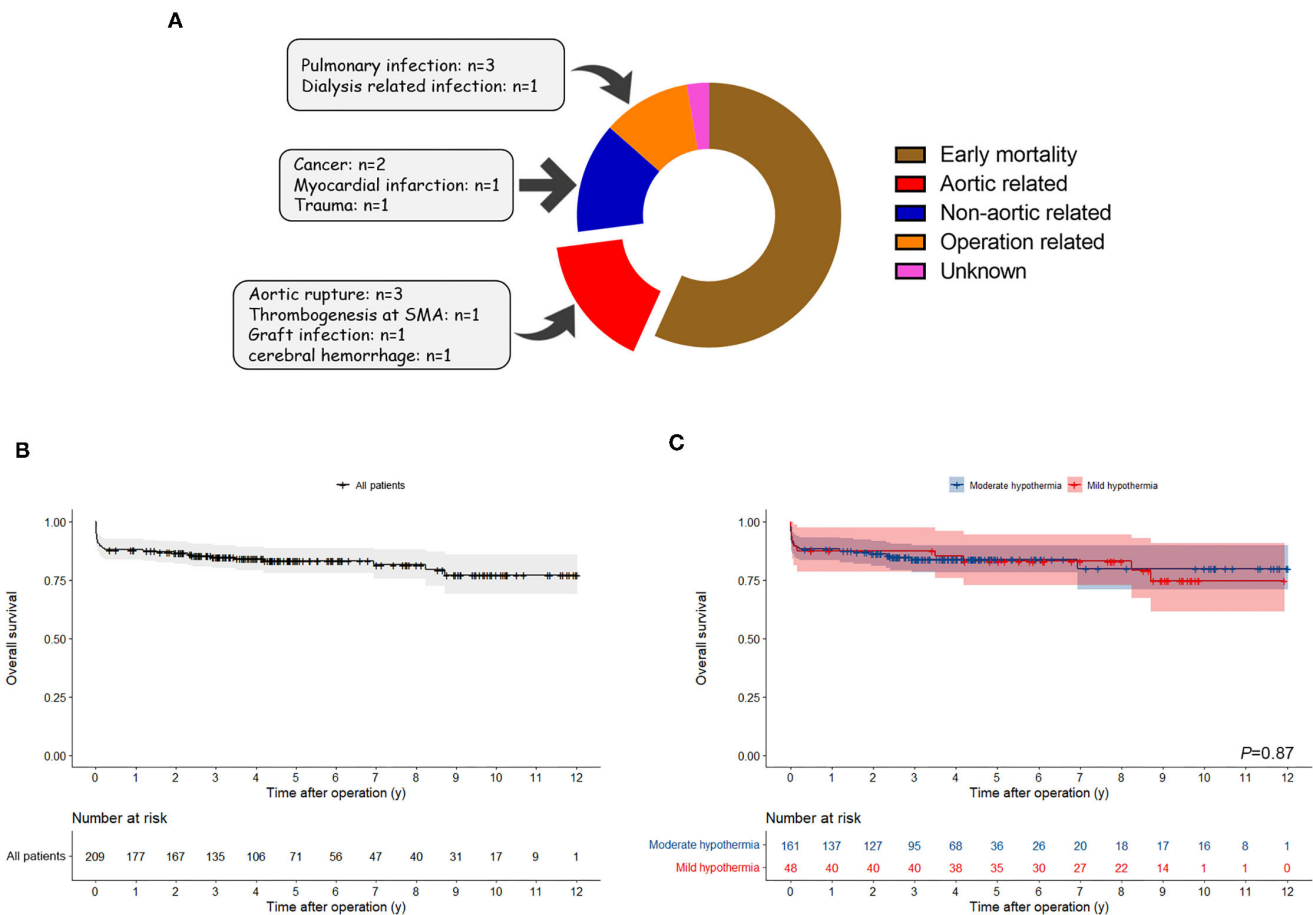
Overall survival analysis. **(A)** The cause of death during follow-up. Late death occurred at 16 patients and 6 of them were aortic related. **(B)** Kaplan–Meier survival curves for overall survival in all patients. **(C)** Kaplan–Meier survival curves for overall survival in the mild and moderate hypothermia group and log-rank test indicated that significant difference was not observed between two groups.

The median follow-up period was 48.3 months (IQR: 28.4–73.7 months, maximum: 144.3 months). For the entire cohort, excluding 21 early death, late death occurred at 16 patients, the causes of death are shown in Figure 3A and the overall survival rate was 88.0, 83.2, and 77.1% at the 1, 5, and 10 years, respectively, which is shown in Figure 3B. As was shown in Figure 3C, the overall survival rate was 87.5, 83.0, and 74.7% at the 1, 5, and 10 years for the mild hypothermia group, which was similar to the 88.2, 83.9, and 79.9% for the moderate hypothermia group (*P* = 0.87).

**Figure 4:**
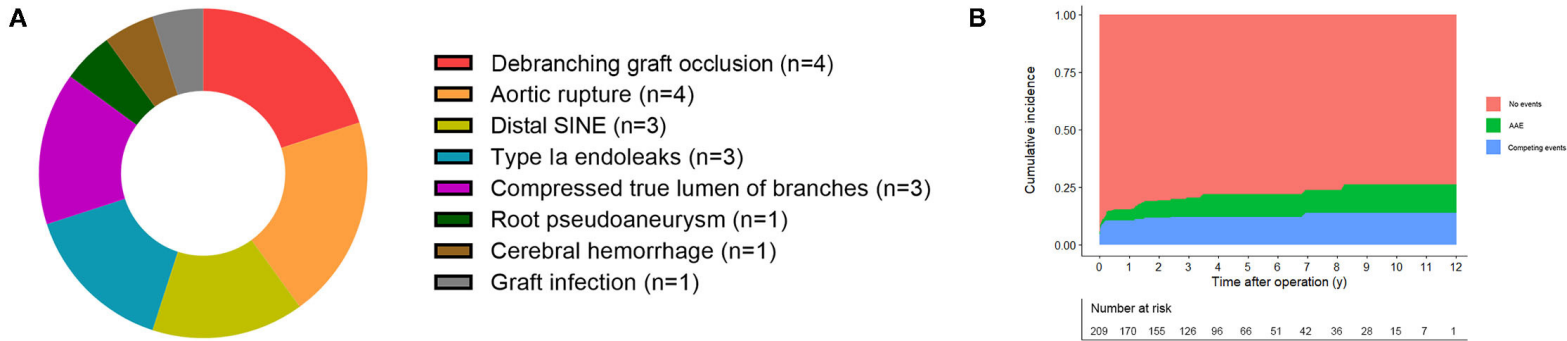
Adverse aortic events analysis. **(A)** The proportion of AAEs in the follow-up. **(B)** Cumulative incidence of AAEs in the presence of death as competing risk. AAEs, adverse aortic events.

Adverse aortic events occurred in 20 patients, three of whom occurred in the hospital, and the detail were shown in Figure 4A. Of those, six aortic-related reintervention were performed, three for the true lumen of branches compressed by the thrombogenesis in false lumen (two occurred in the LCCA and one occurred in the superior mesenteric artery), one for debranching graft occlusion, one for aortic root pseudoaneurysm, and one for distal SINE. In the presence of death as a competing risk, the cumulative incidence of AAEs for the entire cohort was 4.8, 9.9, and 12.1% at the 1, 5, and 10 years, respectively, which was shown in Figure 4B.

#### Long-Term Radiographic Outcomes

**Figure 5:**
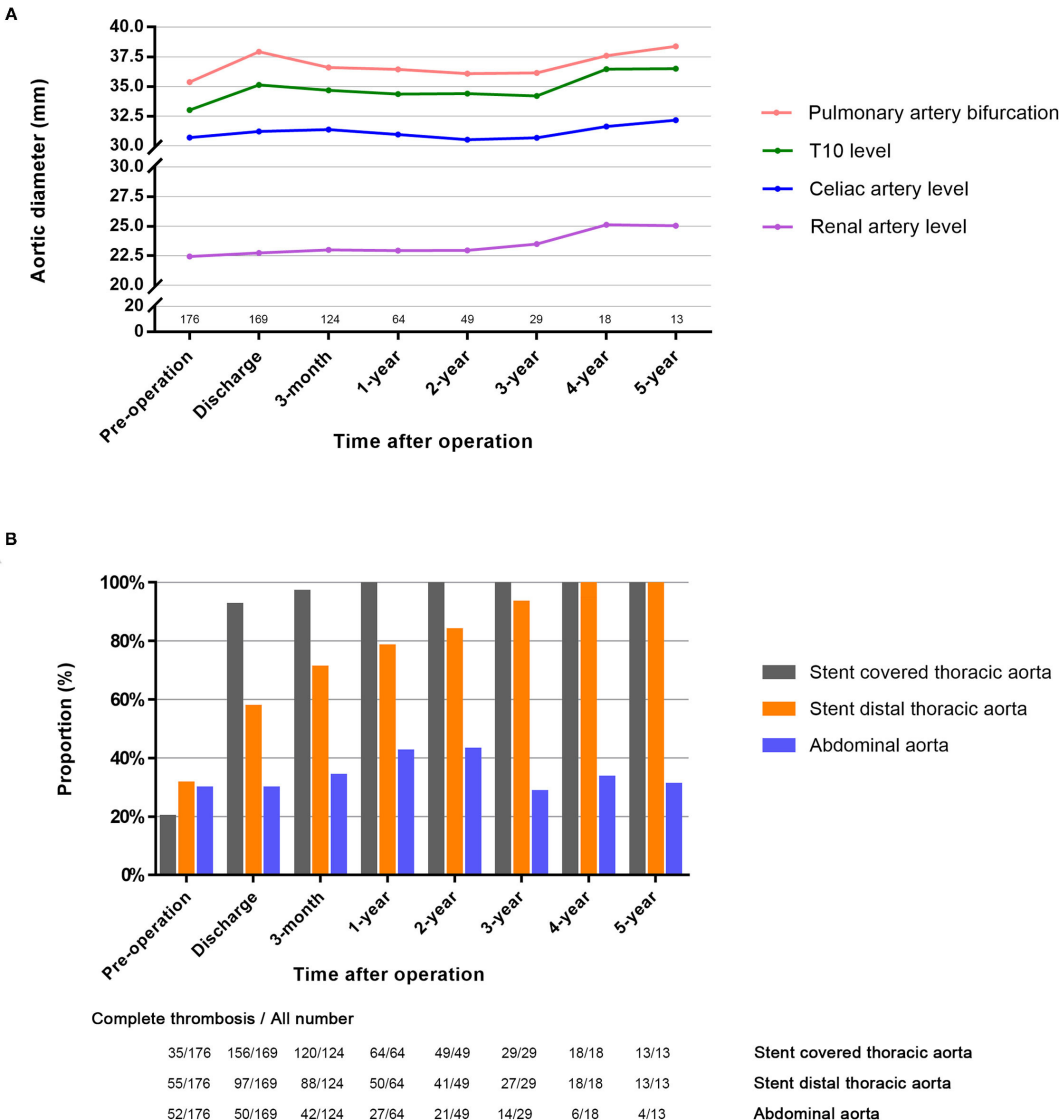
Outcomes of radiographic analysis. **(A)** Aortic diameter changes during the follow-up: the aortic diameter was stable during the follow-up in the descending aorta of pulmonary artery bifurcation, Th10, celiac artery and renal artery level. **(B)** Complete thrombosis of false lumen during the follow-up.

A total of 176 patients were included in analysis, and the median radiographic follow-up period was 4.7 months (maximum: 108 months). The maximum diameters were stable in the aorta of pulmonary artery bifurcation level (35.4 ± 4.2 to 38.4 ± 4.4 mm, *P* = 0.57), Th10 level (33.0 ± 3.7 to 36.5 ± 5.3 mm, *P* = 0.50), celiac artery level (30.7 ± 3.3 to 32.2 ± 5.2 mm, *P* = 0.93), and renal artery level (22.4 ± 3.6 to 25.0 ± 5.0 mm, *P* = 0.71), which was shown in the Figure 5A. False lumen thrombosis results were shown in Figure 5B. In the stent-covered thoracic aorta, complete false lumen thrombosis was 19.9% (35/176) before the operation, and reached 92.3% (156/169) after the operation and reached 100% (64/64) at 1-year follow-up and remained 100% on this level thereafter (*P* < 0.001). In the stent distal thoracic aorta, complete false lumen thrombosis was 31.3% (55/176) before the operation, and reached 57.4% (97/169) after the operation and reached 100% (18/18) at 4-year follow-up (*P* < 0.001). In the abdominal aorta, complete false lumen thrombosis only reached 30.8% (4/13) beyond 5-year follow-up (*P* = 0.41).

### Secondary Endpoints: Early Clinical Outcomes

The overall early composite adverse events morbidity and early mortality were 18.7% (*n* = 39) and 10.0% (*n* = 21). Of those, nine died of multiorgan failure, five died of extensive stroke, four died of postoperative myocardial infarction or LCOS, two died of ischemic intestinal necrosis, and one died of descending aorta sudden rupture. The early composite adverse events morbidity (mild: 14.6%, *n* = 7 vs. moderate: 19.9%, *n* = 32, *P* = 0.41) and early mortality (mild: 10.4%, *n* = 5 vs. moderate: 9.9%, *n* = 16, *P* = 1.00) were similar between the mild and moderate hypothermia groups. Other early clinical outcomes are shown in the Table 3 with detail and no significant difference was observed between the mild and moderate hypothermia groups.

**Table 3:** Early clinical outcomes.

**Variables**	**All patients** **(*n* = 209)**	**Mild hypothermia** **(*n* = 48)**	**Moderate hypothermia** **(*n* = 161)**	***P* Value**
Composite adverse events *n*, (%)	39 (18.7)	7 (14.6)	32 (19.9)	0.41
Early mortality *n*, (%)	21 (10.0)	5 (10.4)	16 (9.9)	1.00
LCOS *n*, (%)	5 (2.4)	0 (0)	5 (3.1)	0.49
Malignant arrhythmia *n*, (%)	3 (1.4)	0 (0)	3 (1.9)	1.00
Stroke *n*, (%)	8 (3.8)	2 (4.2)	6 (3.7)	1.00
Paraplegia *n*, (%)	5 (2.4)	1 (2.1)	4 (2.5)	1.00
hemodialysis *n*, (%)	21 (10.0)	5 (10.4)	16 (9.9)	1.00
Respiratory failure *n*, (%)	14 (6.7)	2 (4.2)	12 (7.5)	0.64
Unplanned reoperation *n*, (%)	12 (5.7)	3 (6.3)	9 (5.6)	1.00
Acute kidney injury *n*, (%)				0.13
Grade 0	60 (28.7)	10 (20.8)	50 (31.1)	
Grade 1	97 (46.4)	23 (47.9)	74 (46.0)	
Grade 2	23 (11.0)	7 (14.6)	16 (9.9)	
Grade 3	29 (13.9)	8 (16.7)	21 (13.0)	
Hepatic dysfunction *n*, (%)	33 (15.8)	6 (12.5)	27 (16.8)	0.48
Temporary neurologic deficit *n*, (%)	39 (18.7)	7 (14.6)	32 (19.9)	0.41
Mechanical ventilation time (hours)	23.0 (15.0~41.5)	21.5 (13.0–38.0)	23.0 (15.5–51.5)	0.16
ICU stays (days)	4 (2–6)	3 (2–6)	4 (2–6)	0.24
Postoperative hospital stays (days)	12 (9–15)	12 (10–15)	12 (9–15)	0.50

*ICU, intensive care unit; LCOS, low cardiac output syndrome*.

The independent risk factors of early clinical outcomes are shown in Table 4. Above all, mild hypothermia was not identified as independent risk factor for any early complications. Multivariable logistic regression analysis found that emergency operation [odds ratio (OR) = 3.47, 95% CI = 1.21–9.92, *P* = 0.020], peripheral arterial disease (OR = 4.94, 95% CI = 1.20–20.40, *P* = 0.027), increasing preoperative Scr (OR = 2.53, 95% CI = 1.05–6.07, *P* = 0.038), and CPB time (OR = 1.02, 95% CI = 1.01–1.03, *P* = 0.005) were identified as independent risk factors for early composite adverse events. Cardiac malperfusion (OR = 5.78, 95% CI = 1.07–31.36, *P* = 0.042), increasing preoperative Scr (OR = 2.82, 95% CI = 1.07–7.42, *P* = 0.035) and CPB time (OR = 1.01, 95% CI = 1.002–1.02, *P* = 0.009) were identified as independent risk factors for early mortality.

**Table 4:** Multivariate logistic regression analysis for early clinical outcomes.

**Risk factor**	**OR**	**95%CI**	***P* value**
Composite adverse events			
Emergency operation	3.47	1.21–9.92	0.020
Peripheral arterial disease	4.94	1.20–20.40	0.027
Increasing preoperative Scr	2.53	1.05–6.07	0.038
CPB time	1.02	1.01–1.03	0.005
Early mortality			
Cardiac malperfusion	5.78	1.07–31.36	0.042
Increasing preoperative Scr	2.82	1.07–7.42	0.035
CPB time	1.01	1.002–1.02	0.009
LCOS			
Cardiac malperfusion	24.74	2.66–229.97	0.005
CPB time	1.01	1.002–1.02	0.048
Hemodialysis			
Female sex	6.22	1.71–22.66	0.006
Age	1.13	1.03–1.23	0.006
Preoperative Scr	1.03	1.01–1.04	<0.001
CPB time	1.01	1.003–1.02	0.005

*CPB, cardiopulmonary bypass; LCOS, low cardiac output syndrome; OR, odds ratio; Scr, serum creatinine*.

## Discussion

The goals of TAAD surgical treatment not only pursue to survive from the operation, but also acquire a long-term survival without adverse events. In the past, excising the primary intimal tear was considered as the priority for the treatment of TAAD. Thus, for the purpose of keeping the patients survive first, ascending aorta replacement featured by less invasion was ever prevailing for TAAD (15). But because of the residual intimal tears and patent false lumen in the aortic arch, the long-term outcome was undesirable (16). Gradually, intervention on the aortic arch received more attention and recognition, so extensive aortic arch repair became widely accepted for TAAD. Both conventional and endovascular total arch surgery for TAAD had their inherent disadvantages. HTAR seemed to avoid the disadvantages of both conventional and endovascular surgeries, and developed to be an alternative for TAAD in our institute, especially for those patients who were unsuitable for conventional TAR.

A study reported that the perioperative mortality of HTAR for complex arch disease (only 20% was TAAD) was 5% from a cohort of 20 patients and another study reported that perioperative mortality was 6% and 5-year overall survival was 63% from a cohort of 34 patents (11, 17). A meta-analysis reported that the perioperative mortality of HTAR for TAAD was 15.1%, higher than 10.8% of all aortic pathology (3). With a 12-year accumulation, our study included almost the largest cohort of HTAR, and focused on a special disease, TAAD, both of which were two main highlights of our study. Besides, the early- and long-term clinical outcomes were both desirable, presenting as 10.0 early mortality, 3.8 stroke morbidity, 10 hemodialysis morbidity, and 83.2% 5-year survival rate.

A previous study reported that in the hemiarch surgery, mild hypothermia (32°C) offered adequate organ protection and achieved low mortality and morbidity (13). But for more invasive extensive arch surgery, attempts to use mild hypothermia (32°C) were rare and the core temperature was usually set at 28°C, even if using some technique intraoperatively to shorten the circulatory arrest time or using bilateral cerebral perfusion (18, 19). HTAR was a concept that avoided distal circulatory arrest and kept the cerebrum continuous perfusion. In the past, one senior surgeon attempted to use mild hypothermia and adjusted the core temperature setting continuously. The highest value of the lowest nasopharyngeal temperature was ever recorded set at 33.7°C, and it was usually set at 32°C, recently. By the statistical analysis, the early clinical outcomes including all early complication of mild hypothermia group were similar to moderate hypothermia group and logistic regression analysis also proved that mild hypothermia was not independent risk factor for early outcomes. Besides, mild hypothermia shortened the CPB and aortic cross-clamping time, which has the potential to reduce the damage from CPB. But we consider that mild hypothermia cannot completely replace the moderate hypothermia, because once unexpected trouble happened intraoperatively and necessitated circulatory arrest, the condition would be very passive. Therefore, we suggest mild hypothermia should be used in patients with low possibility of circulatory arrest and by skilled surgeons.

Because of the difference of medical apparatus and instruments, HTAR reported by some studies was actually TAR with FET procedure performed in our institute. However, HTAR was definitely different from TAR with FET and as an alternative for TAR with FET, especially for those old and high-risk patients. TAR with FET, also called Sun's procedure, achieved desirable outcomes and has been a gold standard on the treatment of TAAD in China (20). One previous study from our institute reported that the age of HTAR group was significantly older than FET group (61.3 vs. 46.7 years), after propensity-score matching, the outcomes of early mortality, early complications and long-term survival in HTAR group was slightly better than TAR with FET groups, but without reaching statistical significance (21). Therefore, performing HTAR in these elderly patients is practical. Previous studies reported that intimal tears located at descending aorta influenced the long-term outcomes (22). But FET or endovascular technique covering the intercostal arteries ostia at the descending aorta had the potential to increase the risk of paraplegia. For HTAR, deploying another stent is easy compared with FET procedure. Meanwhile, in our cohort, the distal position of stents was mostly located at Th7–Th10 and the paraplegia morbidity was only 2.4%. Therefore, for those patients with huge intimal tears on the middle region of descending aorta, HTAR should be a practical and convenient choice.

In our cohort, the 5-year cumulative incidence of AAEs was 9.9%. False lumen thrombosis compressing the true lumen of aortic branches and leading to acute organ ischemia was less common but severe. In our cohort, all of them occurred at early postoperative period, and needed emergency operation and two of them died of extensive target organ ischemia. Distal oversizing and acute dissection were reported associated with distal SINE (23, 24). In our cohort, more than 90% were acute dissection, so the result of three patients occurring distal SINE was acceptable, which was lower than previously reported 4.8%. For preventing distal SINE, we chose deploying another small diameter stent on the distal landing zone before deploying the arch stents to avoid distal oversizing. Debranching graft occlusion was not a common complication, but it occurred at four patients in our cohort. All of them were symptomatic and one of them underwent redo bypass procedure. We considered that carefully adjusting the direction and length of the debranching graft intraoperatively to keep the blood flow patent and appropriate administration of antiatherosclerotic drugs may play an important role.

Aortic remodeling has important influence on the long-term outcomes of TAAD. An European multicenter registry study reported that after FET procedure for TAAD, the thrombosis rate at the stent covered and uncovered regions were 99.3 and 52.6% respectively, whereas the rate at abdominal aorta were low to 13.9%, meanwhile, the diameter at the stent covered descending aorta decreased significantly, at the stent uncovered descending aorta was stable and at the abdominal aorta was increased (25). False lumen persistent patency and intimal tears were reported negatively which were associated with descending aorta remodeling (26). In our cohort, 100% false lumen thrombosis rate and stable diameter in the descending aorta was achieved in the follow-up, which was similar or better than FET technique reported previously. Due to the presence of visceral branch and uncovering intimal tears, patent false lumen at the abdominal aorta was about 60%, but the diameter was stable. Overall, the aortic remodeling of HTAR for TAAD was desirable.

### Limitation

There are some limitations in this study. At first, this study was a retrospective study from a single center, so some inherent bias cannot be excluded. Second, when studying the effect of mild hypothermia on the outcomes, decision on the temperature setting almost completely depended on the operators, which caused that all the operations in the mild hypothermia group were performed by one surgeon and the operations in the moderate hypothermia group were performed by four surgeons. Fortunately, all the four surgeons were senior enough, but the inherent operator difference should not be neglected. Third, because of the pandemic of COVID-19, many patients cannot travel to our institute to undergo CTA examination and they usually chose to underwent CTA examination at local hospital and inquired doctor of our hospital online, which impacted the radiographic follow-up data collection.

## Conclusion

Hybrid total arch replacement without circulatory arrest and prolonged cerebrum ischemia achieved desirable early and long-term clinical outcomes in the surgical treatment of TAAD. Perioperative safety and overall survival of performing HTAR under mild hypothermia for TAAD was similar with moderate hypothermia. After HTAR for TAAD, the aortic diameters were stable in the thoracic and abdominal aorta, and false lumen complete thrombosis in the thoracic aorta reached 100% during the follow-up, which indicated that dissected aorta achieved desirable remodeling after HTAR. All in all, HTAR is a practical treatment for TAAD and provided those elderly or high-risk patients a precious opportunity to receive surgical treatment.

## Data Availability Statement

The original contributions presented in the study are included in the article/Supplementary Material, further inquiries can be directed to the corresponding author.

## Ethics Statement

The studies involving human participants were reviewed and approved by Ethics Committee of Fuwai Hospital. Written informed consent for participation was not required for this study in accordance with the national legislation and the institutional requirements.

## Author Contributions

BZ and XS took the overall responsibility. BZ, XS, and YL were involved in conception and design. BZ, SL, JR, LW, and SZ were involved in the data collection. BZ, SL, and HG gave the statistical analysis. BZ and YL wrote the article. CY, XQ, and XS did the critical revision of the article and were involved in the project administration and resources. HG, JR, LW, and SZ were involved in the analysis and interpretation. All the authors contributed to the article and approved the submitted version.

## Conflict of Interest

The authors declare that the research was conducted in the absence of any commercial or financial relationships that could be construed as a potential conflict of interest.

## Publisher's Note

All claims expressed in this article are solely those of the authors and do not necessarily represent those of their affiliated organizations, or those of the publisher, the editors and the reviewers. Any product that may be evaluated in this article, or claim that may be made by its manufacturer, is not guaranteed or endorsed by the publisher.
