# Supplementary material for: Hybrid Technique on the Total Arch Replacement for Type A Aortic Dissection: 12-year Clinical and Radiographical Outcomes From a Single Center
Source: Front Cardiovasc Med. 2022 Feb 28;9:820653. doi: 10.3389/fcvm.2022.820653 (PMC8918914; doi:10.3389/fcvm.2022.820653)
Supplement: Supplementary file 1 [file Table_1.DOCX]

**Supplemental Table 1 Definition of early clinical outcomes**

| Endpoints | Definition |
| --- | --- |
| Early mortality | Mortality occurred in 30-day after operation or in hospital |
| LCOS | Requiring the support of IABP or ECMO |
| Malignant ventricular arrhythmia | Ventricular tachyarrhythmia, affecting hemodynamics and needing electric cardioversion, but excluding those caused by tamponade and excessive hemorrhage |
| Stroke | New ischemic or hemorrhagic brain injury that was clinically or radiographically evident after the procedure |
| Paraplegia | Myodynamia of lower limb less than or equal to grade 3 (able to resist gravity but not external forces) |
| Hemodialysis | Requiring the support of temporary or continuous hemodialysis, excluding hemodialysis before operation |
| Respiratory failure | Difficult weaning from the ventilator (≥7 days) or requiring tracheal re-intubation or tracheostomy |
| Unplanned reoperation | Unplanned reoperation for hemostasis or severe aortic related complications |
| Acute kidney injury | Categorized by the KDIGO criteria with slight modifications |
| Grade 0 | Serum creatinine increased by less than 1.5 times baseline value and increased by less than 0.3 mg/dL (26.5 μmol/L) |
| Grade 1 | Serum creatinine increased by 1.5–1.9 times baseline value or increased by more than 0.3 mg/dL (26.5 μmol/L) |
| Grade 2 | Serum creatinine increased by 2.0–2.9 times baseline value |
| Grade 3 | Serum creatinine increased by more than 3.0 times baseline value or increase to more than 4.0 mg/dL (353.6 mmol/L) or initiation of hemodialysis |
| Hepatic dysfunction | The peak value of hepatic enzyme exceeds 100IU/L |
| Transient neurologic deficit | Delayed awaking (>72 hours), delirium or hemiparalysis, but without structural alteration on brain imaging examination |
| Mechanical ventilation time | The hours of requiring the support of ventilator |
| ICU stays | The days of requiring to stay in the ICU |
| Postoperative hospital stays | The days from operation to discharge |

ECMO: extracorporeal membrane oxygenation

IABP intra-aortic balloon pump

ICU: intensive care unit

KDIGO: Kidney Disease Improving Global Outcomes

LCOS: Low cardiac output syndrome
